# (*S*)-(+)-Ketamine hydro­chloride

**DOI:** 10.1107/S1600536808021053

**Published:** 2008-07-16

**Authors:** Patrick Hakey, Wayne Ouellette, Jon Zubieta, Timothy Korter

**Affiliations:** a Department of Chemistry, Syracuse University, Syracuse, New York 13244, USA

## Abstract

The crystal structure of the title compound {systematic name: (*S*)-(+)-*N*-[1-(2-chloro­phen­yl)-2-oxocyclo­hexyl]meth­anam­in­ium chloride}, C_13_H_17_ClNO^+^·Cl^−^, was determined at 90 (2) K. The (*S*)-(+)-ketamine hydro­chloride salt is a well known anesthetic compound and is dramatically more potent than its *R* isomer. In the title compound, the cyclo­hexa­none ring adopts a chair conformation with the oxo group in the equatorial orientation. The methyl­amino and 2-chloro­phenyl groups at the 2-position have an equatorial and an axial orientation, respectively. The packing of ions is stabilized by an infinite one-dimensional ⋯Cl⋯H—N—H⋯Cl⋯ hydrogen-bonding network, involving NH_2_^+^ groups as donors and chloride anions as acceptors.

## Related literature

For related literature, see: Chankvetadze *et al.* (2002 ▶); Domino *et al.* (1965 ▶); Marhofer *et al.* (2001 ▶); Mathisen *et al.* (1995 ▶); Pees *et al.* (2003 ▶); Reich & Silvay (1989 ▶); Smirnova *et al.* (1989 ▶); White *et al.* (1985 ▶); Wolff & Winstock (2006 ▶).
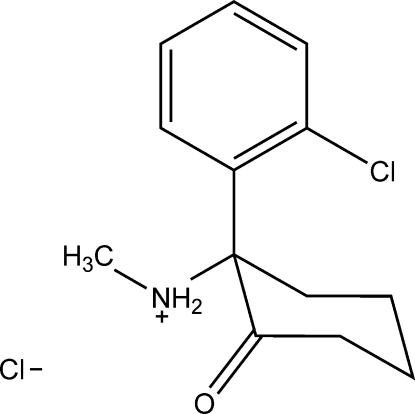


## Experimental

### 

#### Crystal data

C_13_H_17_ClNO^+^·Cl^−^
*M* *_r_* = 274.18
Monoclinic, 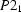

*a* = 8.4338 (4) Å
*b* = 7.0715 (4) Å
*c* = 11.3524 (6) Å
β = 101.875 (1)°
*V* = 662.56 (6) Å^3^
*Z* = 2
Mo *K*α radiation
μ = 0.47 mm^−1^
*T* = 90 (2) K
0.50 × 0.12 × 0.10 mm

#### Data collection

Bruker APEX CCD area-detector diffractometer
Absorption correction: multi-scan (*SADABS*; Sheldrick, 2008 ▶) *T*_min_ = 0.798, *T*_max_ = 0.954
6985 measured reflections
3251 independent reflections
3146 reflections with *I* > 2σ(*I*)
*R*_int_ = 0.020

#### Refinement

*R*[*F* ^2^ > 2σ(*F* ^2^)] = 0.027
*wR*(*F* ^2^) = 0.066
*S* = 1.07
3251 reflections
223 parameters
1 restraint
All H-atom parameters refined
Δρ_max_ = 0.30 e Å^−3^
Δρ_min_ = −0.17 e Å^−3^
Absolute structure: Flack (1983 ▶), 1472 Friedel pairs
Flack parameter: 0.00 (5)

### 

Data collection: *SMART* (Bruker, 2002 ▶); cell refinement: *SAINT* (Bruker, 2002 ▶); data reduction: *SAINT*; program(s) used to solve structure: *SHELXS97* (Sheldrick, 2008 ▶); program(s) used to refine structure: *SHELXL97* (Sheldrick, 2008 ▶); molecular graphics: *CrystalMaker* (Palmer, 2006 ▶); software used to prepare material for publication: *SHELXTL* (Sheldrick, 2008 ▶).

## Supplementary Material

Crystal structure: contains datablocks I, global. DOI: 10.1107/S1600536808021053/bh2181sup1.cif

Structure factors: contains datablocks I. DOI: 10.1107/S1600536808021053/bh2181Isup2.hkl

Additional supplementary materials:  crystallographic information; 3D view; checkCIF report

## Figures and Tables

**Table 1:** Hydrogen-bond geometry (Å, °)

*D*—H⋯*A*	*D*—H	H⋯*A*	*D*⋯*A*	*D*—H⋯*A*
N1—H1*A*⋯Cl2^i^	0.83 (3)	2.39 (3)	3.1359 (15)	151 (2)
N1—H1*B*⋯Cl2	0.869 (19)	2.278 (19)	3.1065 (13)	159.4 (17)

Symmetry code: (i) 
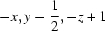
.

**Fig. 1.**
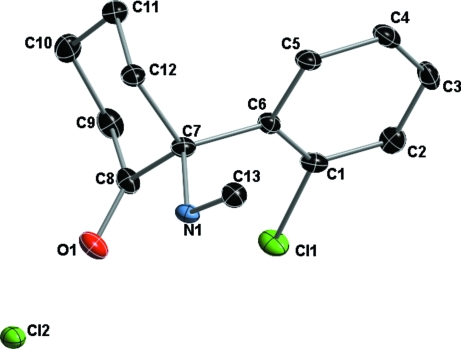
Perspective view of the title salt, with the atom numbering scheme and thermal ellipsoids drawn at 50% probability level. H atoms have been omitted for clarity

**Fig. 2.**
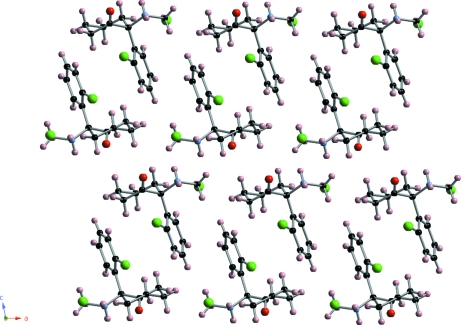
The crystal packing of the title compound viewed along the b axis.

## supplementary crystallographic information

### Comment

The title compound, (*S*)-(+)-ketamine hydrochloride, has been investigated by single-crystal X-ray diffraction at 90 (2) K (Fig. 1 and 2). The use of ketamine has been shown to lead to a state of dissociative anesthesia (Domino *et al.*, 1965). This study focuses on the *S* isomer of the well known anesthetic compound ketamine hydrochloride because it is dramatically more potent than its *R* isomer (White *et al.*, 1985). The pure *S* isomer is available commercially as Ketanest S^TM^, while the racemic mixture, containing both *S* and *R* isomers is available under many names, including: Ketalar^TM^, Ketanest^TM^, and Ketaject^TM^ (Marhofer *et al.*, 2001; Wolff & Winstock, 2006). Both the *S* and *R* isomers of neutral ketamine have been structurally characterized by X-ray diffraction in an earlier investigation (Chankvetadze *et al.*, 2002). The compound is listed as a Schedule III controlled substance by the Federal government of the United States (Wolff & Winstock, 2006). Ketamine hydrochloride was first investigated in the 1960's and is a derivative of the more dangerous psychotomimetic drug, Phencyclidine (Domino *et al.*, 1965). The chirality of Ketamine can be attributed to the C2 position, located on the cyclohexanone ring (Reich & Silvay, 1989). The potency of the drug is dependent upon its conformation, with the S isomer displaying analgesic effects roughly 4 times greater than those displayed by the levorotatory enantiomer *R*-(-)-Ketamine in controlling the pain of postoperative patients (Mathisen *et al.*, 1995). In comparison to the racemic mixture, the *S*-isomer produces analgesic and anesthetic effects with twice the potency of the racemic (White *et al.*, 1985). Increased potency enables the use of a much lower dosage while still producing the required effect, in turn leading to a quicker recovery time from the anesthesia (Pees *et al.*, 2003). The potential advantages of the *S* isomer over the racemic mixture have lead to an increase in clinical use of the enantiomerically pure compound, particularly in Europe (Marhofer *et al.*, 2001). The potency, the increasing clinical usage, and the strong potential for abuse of (*S*)-(+)-Ketamine hydrochloride, provide a need for the complete characterization of this molecule, and its previously unpublished crystal structure. An unequivocal understanding of the solid-state crystal structure of the compound is a necessity for detection and identification methods such as THz vibrational spectroscopy, or solid-state NMR, among others. This study has determined that the compound crystallizes in the monoclinic space group *P*2_1_, with a unit cell volume of 662.56 (6) Å^3^ at 90 K, and *Z* value 2. The complete atomic coordinates have also been determined.

### Experimental

All material used for this work was purchased from Sigma-Aldrich (minimum 99% pure) and used without further purification. The absolute configuration of the enantiomer was verified by measuring the optical rotation of the material using a Jasco DIP-1000 digital polarimeter. The absolute configuration determined from anomalous dispersion-effects is consistent with the expected enantiomer.

### Refinement

H atoms were located in a difference map and refined freely. The C—H and N—H bond lengths range from 0.91 (2) to 1.014 (17) and 0.83 (3) to 0.869 (18) Å, respectively.

### Crystal data

**Table:**

C_13_H_17_ClNO·Cl	*F*_000_ = 288
*M**_r_* = 274.18	*D*_x_ = 1.374 Mg m^−^^3^
Monoclinic, *P*2_1_	Mo *K*α radiation λ = 0.71073 Å
Hall symbol: P 2yb	Cell parameters from 3471 reflections
*a* = 8.4338 (4) Å	θ = 2.5–28.2º
*b* = 7.0715 (4) Å	µ = 0.47 mm^−^^1^
*c* = 11.3524 (6) Å	*T* = 90 (2) K
β = 101.875 (1)º	Rod, colourless
*V* = 662.56 (6) Å^3^	0.50 × 0.12 × 0.10 mm
*Z* = 2	

### Data collection

**Table:**

Bruker APEX CCD area-detector diffractometer	3251 independent reflections
Monochromator: graphite	3146 reflections with *I* > 2σ(*I*)
Detector resolution: 512 pixels mm^-1^	*R*_int_ = 0.020
*T* = 90(2) K	θ_max_ = 28.3º
φ and ω scans	θ_min_ = 1.8º
Absorption correction: multi-scan(SADABS; Sheldrick, 2008)	*h* = −11→11
*T*_min_ = 0.798, *T*_max_ = 0.954	*k* = −9→9
6985 measured reflections	*l* = −15→15

### Refinement

**Table:**

Refinement on *F*^2^	Hydrogen site location: inferred from neighbouring sites
Least-squares matrix: full	All H-atom parameters refined
*R*[*F*^2^ > 2σ(*F*^2^)] = 0.027	*w* = 1/[σ^2^(*F*_o_^2^) + (0.0378*P*)^2^ + 0.0273*P*] where *P* = (*F*_o_^2^ + 2*F*_c_^2^)/3
*wR*(*F*^2^) = 0.066	(Δ/σ)_max_ = 0.001
*S* = 1.07	Δρ_max_ = 0.31 e Å^−^^3^
3251 reflections	Δρ_min_ = −0.17 e Å^−^^3^
223 parameters	Extinction correction: none
1 restraint	Absolute structure: Flack (1983), 1472 Friedel pairs
Primary atom site location: structure-invariant direct methods	Flack parameter: 0.00 (5)
Secondary atom site location: difference Fourier map	

### Fractional atomic coordinates and isotropic or equivalent isotropic displacement parameters (Å^2^)

**Table:**

	*x*	*y*	*z*	*U*_iso_*/*U*_eq_	
Cl2	0.05227 (4)	0.23769 (5)	0.35081 (3)	0.01627 (9)	
Cl1	0.34238 (5)	−0.13679 (5)	0.87212 (4)	0.02248 (10)	
O1	0.38001 (14)	−0.03746 (17)	0.60012 (10)	0.0205 (2)	
N1	0.11098 (16)	0.1291 (2)	0.62148 (11)	0.0132 (3)	
C1	0.27185 (19)	0.0822 (2)	0.90899 (14)	0.0157 (3)	
C2	0.2437 (2)	0.0998 (3)	1.02456 (15)	0.0212 (3)	
C3	0.1889 (2)	0.2691 (3)	1.06202 (16)	0.0239 (4)	
C4	0.1635 (2)	0.4199 (3)	0.98344 (15)	0.0218 (4)	
C5	0.19088 (19)	0.4012 (2)	0.86757 (15)	0.0175 (3)	
C6	0.24628 (16)	0.2315 (3)	0.82673 (13)	0.0140 (3)	
C7	0.26395 (16)	0.2136 (2)	0.69555 (12)	0.0124 (3)	
C8	0.40443 (19)	0.0878 (2)	0.67428 (13)	0.0155 (3)	
C9	0.57016 (19)	0.1543 (2)	0.73576 (15)	0.0176 (3)	
C10	0.59821 (19)	0.3492 (3)	0.68330 (14)	0.0175 (3)	
C11	0.46277 (19)	0.4860 (2)	0.69619 (15)	0.0166 (3)	
C12	0.29711 (19)	0.4052 (2)	0.63815 (14)	0.0142 (3)	
C13	−0.04410 (18)	0.2160 (3)	0.63712 (14)	0.0170 (3)	
H11B	0.471 (2)	0.511 (3)	0.7848 (19)	0.024 (5)*	
H12A	0.294 (2)	0.378 (3)	0.5499 (16)	0.014 (4)*	
H13A	−0.060 (2)	0.181 (3)	0.7166 (17)	0.015 (5)*	
H10B	0.603 (2)	0.334 (3)	0.6003 (17)	0.016 (5)*	
H9A	0.649 (2)	0.055 (3)	0.7199 (18)	0.024 (5)*	
H1B	0.116 (2)	0.140 (3)	0.5461 (17)	0.008 (4)*	
H10A	0.703 (3)	0.397 (3)	0.7211 (17)	0.021 (5)*	
H13B	−0.126 (2)	0.167 (3)	0.5775 (18)	0.021 (5)*	
H9B	0.574 (2)	0.167 (3)	0.8215 (18)	0.018 (5)*	
H5	0.174 (2)	0.503 (3)	0.8174 (19)	0.023 (5)*	
H12B	0.209 (2)	0.490 (3)	0.6419 (16)	0.018 (5)*	
H3	0.166 (2)	0.278 (3)	1.1373 (19)	0.026 (6)*	
H2	0.259 (2)	−0.003 (3)	1.0735 (17)	0.015 (5)*	
H4	0.128 (2)	0.542 (3)	1.0038 (18)	0.022 (5)*	
H13C	−0.038 (2)	0.351 (3)	0.6318 (16)	0.018 (5)*	
H1A	0.105 (3)	0.015 (4)	0.6352 (18)	0.024 (5)*	
H11A	0.483 (3)	0.601 (4)	0.6570 (18)	0.029 (6)*	

### Atomic displacement parameters (Å^2^)

**Table:**

	*U*^11^	*U*^22^	*U*^33^	*U*^12^	*U*^13^	*U*^23^
Cl2	0.02035 (17)	0.01438 (16)	0.01376 (16)	0.00042 (14)	0.00279 (12)	−0.00059 (13)
Cl1	0.0302 (2)	0.01408 (17)	0.02266 (19)	0.00260 (16)	0.00414 (15)	0.00452 (14)
O1	0.0253 (6)	0.0180 (6)	0.0188 (6)	0.0042 (5)	0.0059 (5)	−0.0035 (5)
N1	0.0159 (6)	0.0117 (6)	0.0117 (6)	0.0003 (5)	0.0019 (5)	−0.0008 (5)
C1	0.0155 (7)	0.0150 (7)	0.0163 (7)	−0.0006 (6)	0.0020 (6)	0.0008 (6)
C2	0.0197 (8)	0.0285 (9)	0.0145 (7)	−0.0054 (7)	0.0016 (6)	0.0048 (7)
C3	0.0211 (8)	0.0358 (11)	0.0159 (8)	−0.0058 (7)	0.0064 (6)	−0.0049 (7)
C4	0.0192 (8)	0.0261 (9)	0.0203 (8)	−0.0013 (6)	0.0048 (6)	−0.0092 (7)
C5	0.0170 (7)	0.0169 (8)	0.0180 (7)	0.0013 (6)	0.0022 (6)	−0.0013 (6)
C6	0.0129 (6)	0.0164 (7)	0.0126 (6)	−0.0008 (6)	0.0026 (5)	−0.0003 (6)
C7	0.0130 (6)	0.0126 (7)	0.0113 (6)	0.0003 (6)	0.0015 (5)	0.0006 (6)
C8	0.0192 (8)	0.0145 (7)	0.0135 (7)	0.0044 (6)	0.0053 (6)	0.0035 (6)
C9	0.0148 (7)	0.0197 (8)	0.0183 (8)	0.0046 (6)	0.0038 (6)	0.0024 (6)
C10	0.0159 (7)	0.0200 (8)	0.0171 (7)	−0.0001 (6)	0.0044 (6)	0.0002 (6)
C11	0.0171 (7)	0.0143 (7)	0.0186 (8)	−0.0001 (6)	0.0042 (6)	0.0012 (6)
C12	0.0153 (7)	0.0127 (7)	0.0146 (7)	0.0003 (5)	0.0028 (5)	0.0013 (5)
C13	0.0149 (7)	0.0163 (8)	0.0197 (7)	−0.0001 (6)	0.0033 (5)	−0.0022 (7)

### Geometric parameters (Å, °)

**Table:**

Cl1—C1	1.7403 (16)	C7—C8	1.540 (2)
O1—C8	1.210 (2)	C7—C12	1.553 (2)
N1—C13	1.488 (2)	C8—C9	1.503 (2)
N1—C7	1.5108 (19)	C9—C10	1.539 (2)
N1—H1B	0.869 (18)	C9—H9A	1.01 (2)
N1—H1A	0.83 (3)	C9—H9B	0.97 (2)
C1—C2	1.386 (2)	C10—C11	1.527 (2)
C1—C6	1.397 (2)	C10—H10B	0.957 (18)
C2—C3	1.382 (3)	C10—H10A	0.96 (2)
C2—H2	0.91 (2)	C11—C12	1.528 (2)
C3—C4	1.378 (3)	C11—H11B	1.01 (2)
C3—H3	0.92 (2)	C11—H11A	0.96 (2)
C4—C5	1.388 (2)	C12—H12A	1.014 (17)
C4—H4	0.96 (2)	C12—H12B	0.96 (2)
C5—C6	1.401 (2)	C13—H13A	0.971 (19)
C5—H5	0.91 (2)	C13—H13B	0.93 (2)
C6—C7	1.5321 (19)	C13—H13C	0.96 (2)
			
C13—N1—C7	116.17 (12)	C9—C8—C7	114.74 (13)
C13—N1—H1B	107.8 (12)	C8—C9—C10	107.60 (13)
C7—N1—H1B	107.7 (12)	C8—C9—H9A	106.5 (12)
C13—N1—H1A	106.8 (15)	C10—C9—H9A	113.3 (12)
C7—N1—H1A	111.3 (15)	C8—C9—H9B	109.6 (12)
H1B—N1—H1A	106.7 (19)	C10—C9—H9B	109.0 (12)
C2—C1—C6	122.11 (15)	H9A—C9—H9B	110.8 (16)
C2—C1—Cl1	116.19 (13)	C11—C10—C9	110.66 (13)
C6—C1—Cl1	121.69 (12)	C11—C10—H10B	110.3 (11)
C3—C2—C1	120.18 (16)	C9—C10—H10B	108.8 (12)
C3—C2—H2	121.6 (12)	C11—C10—H10A	111.8 (13)
C1—C2—H2	118.2 (12)	C9—C10—H10A	110.1 (13)
C4—C3—C2	119.23 (15)	H10B—C10—H10A	105.0 (16)
C4—C3—H3	121.0 (14)	C10—C11—C12	110.94 (14)
C2—C3—H3	119.7 (14)	C10—C11—H11B	108.0 (12)
C3—C4—C5	120.40 (16)	C12—C11—H11B	111.3 (12)
C3—C4—H4	123.7 (13)	C10—C11—H11A	106.8 (14)
C5—C4—H4	115.9 (13)	C12—C11—H11A	110.4 (13)
C4—C5—C6	121.81 (16)	H11B—C11—H11A	109.3 (17)
C4—C5—H5	118.8 (13)	C11—C12—C7	111.90 (12)
C6—C5—H5	119.4 (13)	C11—C12—H12A	109.5 (10)
C1—C6—C5	116.27 (13)	C7—C12—H12A	106.2 (12)
C1—C6—C7	123.69 (15)	C11—C12—H12B	113.2 (13)
C5—C6—C7	119.89 (14)	C7—C12—H12B	108.5 (12)
N1—C7—C6	109.33 (11)	H12A—C12—H12B	107.2 (15)
N1—C7—C8	106.29 (12)	N1—C13—H13A	107.3 (11)
C6—C7—C8	115.60 (12)	N1—C13—H13B	107.4 (12)
N1—C7—C12	108.57 (11)	H13A—C13—H13B	111.0 (17)
C6—C7—C12	113.44 (13)	N1—C13—H13C	110.3 (11)
C8—C7—C12	103.13 (11)	H13A—C13—H13C	109.3 (16)
O1—C8—C9	124.09 (15)	H13B—C13—H13C	111.5 (17)
O1—C8—C7	120.38 (14)		
			
C6—C1—C2—C3	−0.2 (2)	C5—C6—C7—C8	146.41 (14)
Cl1—C1—C2—C3	179.44 (13)	C1—C6—C7—C12	−157.03 (14)
C1—C2—C3—C4	−0.3 (3)	C5—C6—C7—C12	27.58 (18)
C2—C3—C4—C5	0.7 (3)	N1—C7—C8—O1	6.73 (18)
C3—C4—C5—C6	−0.7 (3)	C6—C7—C8—O1	128.23 (15)
C2—C1—C6—C5	0.2 (2)	C12—C7—C8—O1	−107.40 (16)
Cl1—C1—C6—C5	−179.37 (11)	N1—C7—C8—C9	177.01 (12)
C2—C1—C6—C7	−175.34 (14)	C6—C7—C8—C9	−61.50 (17)
Cl1—C1—C6—C7	5.1 (2)	C12—C7—C8—C9	62.88 (15)
C4—C5—C6—C1	0.2 (2)	O1—C8—C9—C10	107.30 (17)
C4—C5—C6—C7	175.93 (14)	C7—C8—C9—C10	−62.57 (16)
C13—N1—C7—C6	48.63 (18)	C8—C9—C10—C11	55.38 (17)
C13—N1—C7—C8	174.05 (13)	C9—C10—C11—C12	−55.57 (17)
C13—N1—C7—C12	−75.59 (16)	C10—C11—C12—C7	59.27 (17)
C1—C6—C7—N1	81.64 (16)	N1—C7—C12—C11	−171.44 (12)
C5—C6—C7—N1	−93.75 (17)	C6—C7—C12—C11	66.81 (16)
C1—C6—C7—C8	−38.2 (2)	C8—C7—C12—C11	−58.97 (15)

### Hydrogen-bond geometry (Å, °)

**Table:**

*D*—H···*A*	*D*—H	H···*A*	*D*···*A*	*D*—H···*A*
N1—H1A···Cl2^i^	0.83 (3)	2.39 (3)	3.1359 (15)	151 (2)
N1—H1B···Cl2	0.869 (19)	2.278 (19)	3.1065 (13)	159.4 (17)

Symmetry codes: (i) −*x*, *y*−1/2, −*z*+1.
